# Sonographic and Clinical Features of Upper Extremity Deep Venous Thrombosis in Critical Care Patients

**DOI:** 10.1155/2012/489135

**Published:** 2012-05-13

**Authors:** Michael Blaivas, Konstantinos Stefanidis, Serafim Nanas, John Poularas, Mitchell Wachtel, Rubin Cohen, Dimitrios Karakitsos

**Affiliations:** ^1^Department of Emergency Medicine, North Side Hospital Forsyth, Cumming, GA 30041, USA; ^2^Radiology and 1st Critical Care Departments, Evangelismos University Hospital, 10676 Athens, Greece; ^3^Intensive Care Unit, General State Hospital of Athens, 10676 Athens, Greece; ^4^Department of Biostatistics, Texas Tech University, Lubbock, TX 79409, USA; ^5^Division of Pulmonary and Critical Care Medicine, Hofstra North Shore-LIJ School of Medicine, The Long Island Jewish Medical Center, New York, NY 11549, USA

## Abstract

*Background-Aim*. Upper extremity deep vein thrombosis (UEDVT) is an increasingly recognized problem in the critically ill. We sought to identify the prevalence of and risk factors for UEDVT, and to characterize sonographically detected thrombi in the critical care setting. *Patients and Methods*. Three hundred and twenty patients receiving a subclavian or internal jugular central venous catheter (CVC) were included. When an UEDVT was detected, therapeutic anticoagulation was started. Additionally, a standardized ultrasound scan was performed to detect the extent of the thrombus. Images were interpreted offline by two independent readers. *Results*. Thirty-six (11.25%) patients had UEDVT and a complete scan was performed. One (2.7%) of these patients died, and 2 had pulmonary embolism (5.5%). Risk factors associated with UEDVT were presence of CVC [(odds ratio (OR) 2.716, *P* = 0.007)], malignancy (OR 1.483, *P* = 0.036), total parenteral nutrition (OR 1.399, *P* = 0.035), hypercoagulable state (OR 1.284, *P* = 0.045), and obesity (OR 1.191, *P* = 0.049). Eight thrombi were chronic, and 28 were acute. We describe a new sonographic sign which characterized acute thrombosis: a double hyperechoic line at the interface between the thrombus and the venous wall; but its clinical significance remains to be defined. *Conclusion*. Presence of CVC was a strong predictor for the development of UEDVT in a cohort of critical care patients; however, the rate of subsequent PE and related mortality was low.

## 1. Introduction

Upper extremity deep venous thrombosis (UEDVT) may be underdiagnosed as imaging of these vessels is not a routine part of pulmonary embolism (PE) investigation [1]. Moreover, PE is thought to occur at low rates (7 to 9%) in patients with UEDVT [1–3]. The clinical significance of UEDVT remains uncertain and much variability in reported treatment [4]. Nevertheless, current guidelines recommend that UEDVT should be treated similarly to lower extremity deep venous thrombosis [5]. In various series, 35 to 75% of patients who have upper extremity, neck, or torso central venous catheters (CVCs) develops thrombosis, with 75% being asymptomatic [6–10]. CVCs have been increasingly used in the intensive care unit (ICU) hence there is rationale to further investigated UEDVT [11–15]. CVC-associated UEDVT may be related to the material the catheter is made of and its diameter [11–17]. Other commonly reported risk factors for development of UEDVT are malignancy and thrombophilia. Less frequently reported risk factors include an obstructing tumor, pregnancy, and estrogen use [1, 18–21]. However, it is difficult to find extensive data on the incidence and clinical characteristics of UEDVT in the ICU [9, 10, 12].

The situation is further complicated by the use of different imaging techniques to diagnose UEDVT such as radionuclide scanning, ultrasound, magnetic resonance imaging (MRI), computed tomography (CT), and contrast venography. Venography remains the reference standard but cannot be used readily in the critically ill and has a small incidence of complications [10, 22]. Ultrasonography is considered the initial imaging test of choice as it can exclude deep venous thrombosis and identify proximal venous obstruction [1, 3, 8–11, 21, 23]. The advantages of this test include its noninvasiveness, portability, lack of ionizing radiation, and high sensitivity and specificity [23].

In this study, we aimed to clarify the clinical uncertainties and risk factors associated with the diagnosis and significance of UEDVT by retrospectively analyzing ultrasound data derived from a cohort of critical care patients. Moreover, we analyzed the sonographic features of detected thromboses in order to assess thrombus age.

## 2. Materials and Methods

We extracted data from the archives of previously registered trials, which were conducted by our team and concerned subclavian (SCV) and internal jugular vein (IJV) ultrasound-guided cannulation (ISRCTN-61258470) [24, 25]. The present study was approved by the General State Hospital of Athens ethical committee. Three hundred and twenty critical care patients, who were hospitalized in a multipurpose intensive care unit (ICU) from 2006 to 2012, and in whom complete sonographic records were available for retrieval, were enrolled. All patients were sedated and mechanically ventilated (Servo-I ventilator, Maquet Inc., Bridgewater, NJ, USA). All patients were routinely scanned before, during and after ultrasound-guided IJV and SCV cannulation by means of a portable HD11 XE ultrasound machine (Philips, Andover, MA, USA) equipped with a high-resolution 7.5–12 MHz transducer, as described in detail elsewhere [1, 2]. When an UEDVT was identified, a complete scanning protocol was initiated [23, 26]. In brief, the IJV was examined from the level of the mandible to the point at which it traveled under the clavicle. The junction of the SCV and IJV originating at the innominate vein is difficult to visualize, therefore Doppler was utilized to provide indirect information regarding the patency of the veins in this area. Next, the SCV was followed in the direction of the clavicle distally until it anatomically changed to the axillary vein, which in turn was followed in the direction of the upper arm where the brachial vein was identified. The latter was followed distally until the junction of the radial and ulnar veins, which in turn were followed until the region of the wrist. Thus, a complete assessment of the deep veins of the upper extremity and torso was completed. Ultrasound scanning included utilization of two-dimensional (2D) scanning with compression testing and Color-Doppler modes. Venous thromboses were identified according to American College of Radiology criteria [23].

All ultrasound data were stored in a computerized off-line system. Sonographic images were reviewed retrospectively by one independent radiologist and one intensivist trained in vascular ultrasound, both of whom were blinded to the subjects' clinical characteristics. When a visible intraluminal thrombus was identified, several of its characteristics were evaluated to determine its relative age. Sonographic features suggesting chronic thrombosis were a contracted venous segment, thrombus adherence to the venous wall, hyperechoic and heterogeneous appearance of the clot, partial recanalization of the vessel, and presence of venous collaterals. Features suggestive of acute thrombosis were venous distention, a partially compressible or noncompressible lumen, hypoechoic, homogeneous appearance of clots, and presence of free floating thrombi [26–35]. UEDVT was characterized either as spontaneous if no intravascular catheters were related to the thrombus or as CVC associated [1, 8, 9, 21, 24]. The segmental location of thrombosis was analyzed according to the affected veins (IJV, SCV, innominate, axillary and brachial veins). All ultrasound images were analytically reviewed to investigate whether any other sonographic findings related to thrombosis age existed.

Clinical parameters included: patient age, diagnosis upon admission, days of hospitalization, CVC insertion location, type of CVC (triple lumen, double-lumen catheter used for hemodialysis), other indwelling vascular devices (i.e., pacemakers), administration of total parenteral nutrition (TPN), known anatomic vascular anomaly and hypercoagulable disorder, untreated coagulopathy, increased (≥35 kg/m^2^) body mass index (BMI), and known malignancy [1, 2, 8, 12, 14–21]. Use of prophylactic treatment with low molecular weight heparin (LMWH) and subsequent incidence of PE and ICU death was investigated [1–3]. Moreover, we analyzed the sonographic features of recorded thrombosis in an effort to assess the relative age of the thrombus.

## 3. Statistical Analysis

Continuous data were expressed as mean ± standard deviation (SD). The student's *t*-test or Fisher's exact test was used as appropriate to compare group means for patient data. A two-sided *P* value of <0.05 was considered significant. Agreement between the two observers in the evaluation of sonographic data was evaluated by Cohen's weighted *κ*, with 2.5th and 97.5th percentiles of 5,000 bootstrap replicates estimated, using 95% confidence intervals [13]. Multivariate logistical regression in determining potential risk factors facilitating the development of UEDVT, as well as all other statistical analyses, was performed using the R2.10.1 statistical package (R Development Core Team, 2009. R: A language and environment for statistical computing. R Foundation for Statistical Computing, Vienna, Austria).

## 4. Results

Demographic and clinical characteristics of the total study population are presented in Table 1. Thirty-six cases of UEDVT were recorded out of 320 patients reviewed (11.25%). The vast majority of patients in this cohort were trauma victims. All patients had CVC inserted and received prophylactic treatment with LMWH (Table 1).

**Figure 1:**
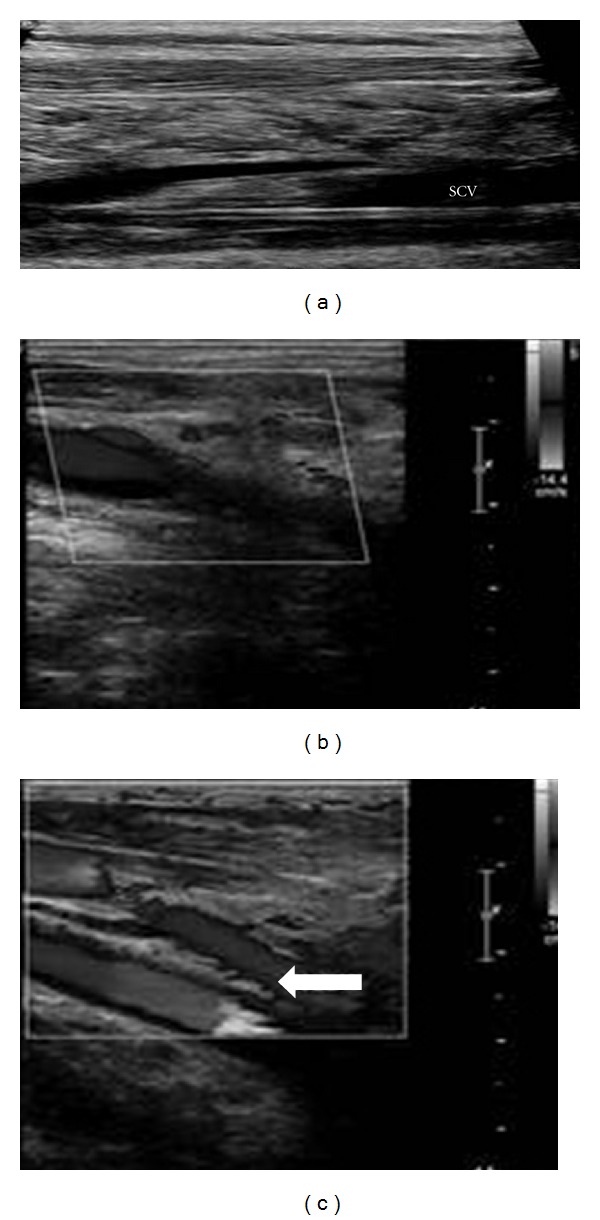
Left subclavian vein catheter-associated chronic thrombosis with partial recanalization (a); proximal right internal jugular vein (b) and ipsilateral subclavian vein (c) with associated collateral flow (arrow) in a patient with chronic spontaneous thrombosis. SCV: Subclavian Vein.

**Figure 2:**
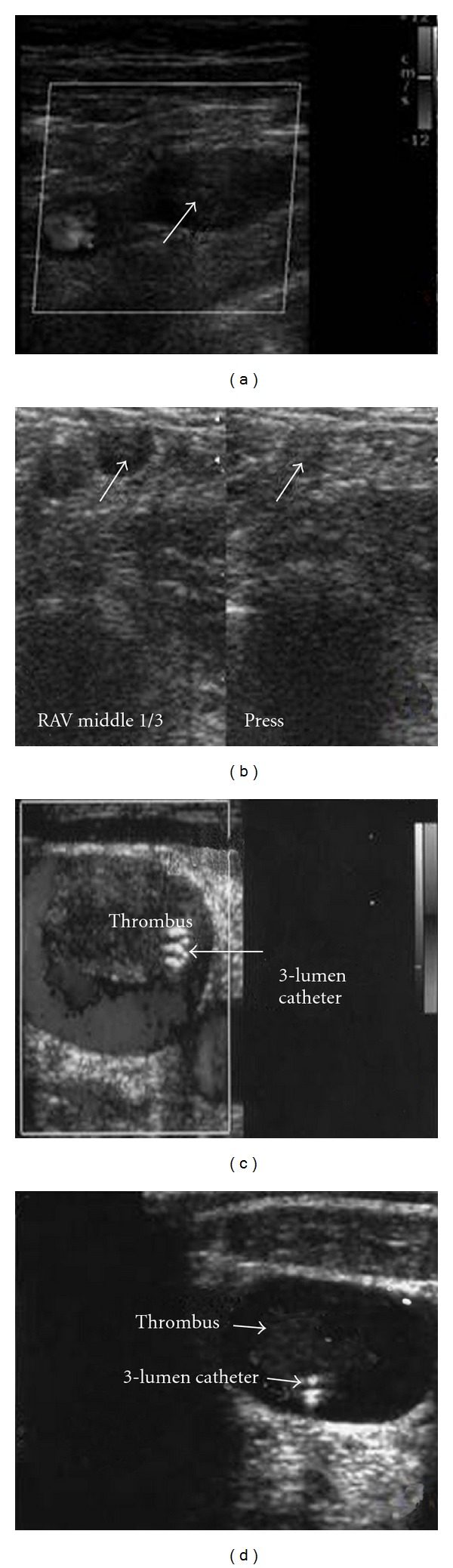
Incompressible proximal right and medial left axillary veins (arrows) in two cases of spontaneous acute thrombosis, respectively ((a), (b)); two cases of catheter-associated thrombosis of the right internal jugular vein with fresh clots obstructing almost totally the venous lumen (c); one of the three lumens of the catheter (in this case delivering total parenteral nutrition, (d)).

Clinical and sonographic characteristics of the 36 cases identified with UEDVT are shown in Table 2. UEDVT was most commonly observed in the SCV and IJV sites, while the number of veins involved was usually 1 to 3. The vast majority of UEDVTs recorded were CVC-associated thromboses (91.7%). Acute thromboses (77.8%) were more commonly observed compared to chronic ones (Table 2) (Figures 1 and 2). UEDVT was mainly symptomatic (55.6%) presenting with edema (20/20) and erythema (5/20) of the affected extremity; however, some asymptomatic cases were noted (Table 2). Other factors that also predisposed to thromboses such as obesity, TPN, and malignancy; these parameters are presented in Table 2. Six of the 9 thromboses associated with TPN were catheter associated. Eight cases of thrombophilia were recorded in patients with UEDVT, which were attributed to mutations of factor V and prothrombin gene (6 cases) and to protein C and S deficiency (2 cases), following laboratory investigation. Notably, only two cases of subsequent PE (5.5%) and one death were recorded in patients with UEDVT (Table 2). All critical care patients with UEDVT received full anticoagulation treatment (unfractionated or LMWH) with no side effects noted.

**Figure 3:**
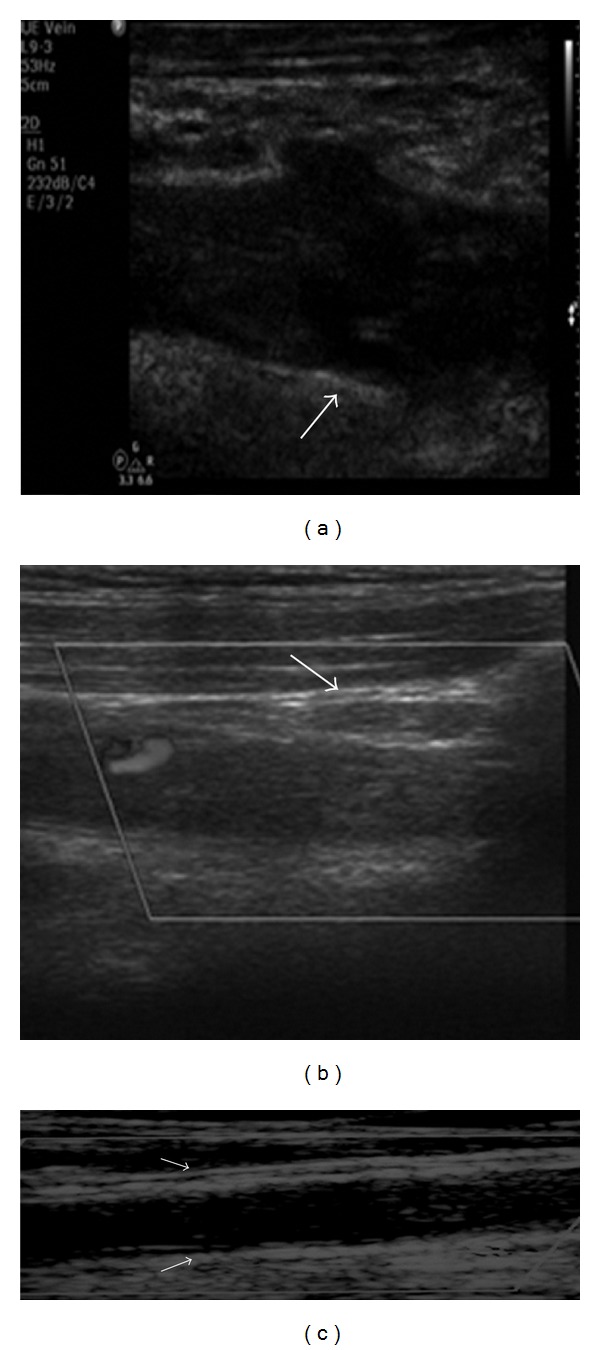
Double hyperechoic line along fresh thrombus/wall interface (arrows) in limited segments of the subclavian vein ((a), (b)) and in extended segments of the left brachial vein (panoramic view with zoom, (c)).

Table 3 presents the typical sonographic features of UEDVT as registered by the two independent observers. Clot adherence to the venous wall, partial recanalization of the lumen, and presence of venous collaterals was associated with chronic thrombosis; while free-floating thrombi with echolucent and homogeneous appearance, lack of compressibility and distended veins were observed in acute thrombosis (Figures 1 and 2). Notably, in 20 out of 28 cases of acute thrombosis a double hyperechoic line along the thrombus and wall interface was identified (Table 3, Figure 3). The overall agreement between the two observers who reviewed the sonographic findings was significant (*κ* = 0.88, 95% confidence intervals by bootstrap analysis = 0.85–0.93, *P* < 0.02).

Multivariate logistic regression analysis (Table 4) identified that insertion of CVC (both triple lumen and double-lumen catheters for hemodialysis), administration of TPN; presence of malignancy, presence of thrombophilia as well as body mass index ≥35 kg/m^2^ were all significantly correlated with the occurrence of UEDVT (all *P* < 0.05). Notably, insertion of CVC was the factor with the strongest effect upon the incidence of UEDVT (odds ratio = 2.716, 95% confidence intervals = 2.312–2.911; *P* = 0.007). 

## 5. Discussion

A rate of 11.25% of UEDVT in patients being examined for CVC placement was detected in this study, consistent with previously published series [1, 2, 9, 20, 21, 36]. Ultrasound was able to diagnose UEDVT and a high agreement was registered between the two independent observers as previously suggested [8, 11, 22, 23, 26, 28–30, 32, 36]. The sensitivity of Doppler sonography in the diagnosis for UEDVT has been reported to range from 78% to 100% and its specificity from 82% to 100% in various series [8–11, 22, 23, 32, 36]. Our results add to the prior literature on detection of UEDVT in ICU patients.

The subsequent rate of PE was on the low end of previously published data [1–4, 12, 20, 21, 36, 37]. The reasons for this are not entirely clear; however, we note that in this study, all patients with UEDVT were fully anticoagulated as per recommended guidelines [5]. Notably, in the study of Mustafa et al., all patients with symptomatic UEDVT received anticoagulant therapy and none developed PE [9]. However, despite guideline recommendations, prescribing full anticoagulation for UEDVT remains controversial [1–3].

The highest risk for UEDVT was having a CVC. Of note, these were all critically ill patients who were being examined for CVC insertion. Risk factors have been established for catheter-related DVT which include catheter material, diameter, and position of the catheter. Clinical and in vitro studies have demonstrated that both polyurethane and silicone catheters are associated with a lower rate of catheter-related DVT as compared with polyethylene or Teflon-coated catheters [16, 17]. In our study all CVCs were polyurethane.

The pathogenesis of thrombosis is multifactorial and ICU patients may have a higher incidence of risk factors than the general population. We confirmed previously published data which suggested that obesity, malignancy, a hypercoagulable state, and administration of TPN are potential risk factors for UEDVT [9, 12, 14, 15, 37, 38]. Obesity may predispose to DVT via several mechanisms, including the physical effects of body fat inhibiting venous return as well as endocrine changes and changes in signaling molecules. Obesity is a proinflammatory, prothrombotic, and hypofibrinolytic state with increased concentrations of coagulation factors and plasminogen activator inhibitor-1 [39]. Out of the 9 patients who had thromboses associated with TPN, 6 were catheter associated. Infusion of TPN may be irritating to veins causing vascular injury and inflammation that is prothrombotic [40].

It is increasingly noted that venous thrombosis is associated with an inflammatory response which plays an essential role in both formation and resolution. This could explain why ICU patients are at higher risk of thrombosis. Thrombus generation is dependent on adhesion molecules such as the selectin family. These molecules are critical for recruitment and attachment of inflammatory cells and fibrin deposition within the thrombus. The mechanism of delivery of the components necessary for thrombus formation is via microparticles shed from the plasma membrane of various cells. Leukocytes and cytokines associated with inflammation are also essential to angiogenesis and fibrinolysis of thrombus resolution. Outlining the inflammatory mechanisms involved in the genesis and resolution response may lead to potential treatments for venous thromboembolism. Current anticoagulation therapies primarily that prevent thrombus propagation are associated with bleeding risk, and do not directly modulate the associated inflammation [41–46].

We also found that the most common location of UEDVT was in the IJV and SCV sites as observed by others [1, 2, 7, 9, 10, 12, 18, 20, 36, 37] and that UEDVT may involve several venous segments [1, 2, 9, 10, 12, 37]. Since femoral catheters were not placed in this study, our data do not challenge prior evidence that femoral CVCs have the highest thrombotic risk [47].

In addition to detecting thromboses, ultrasound facilitated the estimation of the relative age of clots in this cohort. Cases of acute thrombosis were more commonly observed than cases of chronic thrombosis. Clot adherence to the venous wall, partial recanalization of the lumen, and presence of venous collaterals were commonly observed in chronic thrombosis; while free-floating thrombi with echolucent and homogeneous appearance as well as noncompressible, distended veins were observed in acute thrombosis, respectively [26–30].

We also described a new sonographic sign in 2D images that was present in 20 out of 28 cases of acute thrombosis: a double hyperechoic line at the interface between the thrombus and the venous wall. Histology studies show that a smooth coat of fibrin lines the external surface of an acute thrombus [48]. The latter are three-dimensional networks of fibrin fibers stabilized by factor XIIIa. Fibrin fibers are long, thin fibers that easily bend rather than stretch; however, fibrin itself is very dense and stiff thus exhibiting high acoustic impedance [26–30]. We speculate that this double hyperechoic line might represent fibrin fibers coating acute clots. If confirmed in other studies, this finding may assist in determining thrombus age. Recently, Rubin et al. suggested that sonographic elasticity imaging, a technique measuring tissue hardness, can discriminate between acute and chronic thrombi [49, 50].

There were several limitations in this study. First, the study was performed retrospectively solely in patients receiving a CVC and may not be extrapolated to all critically ill patients. Second, ultrasound is an operator-dependent technique, and the amount of training required to become facile at upper extremity DVT examination is unknown. Third, there were a small number of cases of UEDVT, confirming previously reported studies. The small numbers precluded subgroup analyses. However, despite the small numbers we were able to find various parameters that were significantly related to thrombosis. Fourth, our cohort consisted of a high percentage of trauma patients which may affect the generalizability of our findings. Further prospective studies would be required to confirm that a strategy of universal anticoagulation is correct for all ICU patients with this problem.

## 6. Conclusions

The present study characterized the incidence of UEDVT in a mixed ICU population. We confirmed risk factors associated with UEDVT including presence of a CVC, BMI > 35, a hypercoagulable state, malignancy, and use of TPN. We have fully characterized the locations, extent, and ultrasound findings of UEDVTs in an ICU population. In this study, a clinical strategy of universal anticoagulation led to favorable outcomes. We also describe a new ultrasound finding of acute thrombosis: a double hyperechoic line at the interface between the thrombus and the venous wall. Further studies are required to document the utility of this sign as well as the best methods to diagnose and treat UEDVT in ICU patients.

## Figures and Tables

**Table 1:** Baseline characteristics of the study population.

Characteristics	Patients *n* = 320
Age (years)	51 ± 15.5
Gender (male/female ratio)	0.52 ± 0.4
APACHE II score	20.2 ± 3.1
Diagnosis upon admission	
Trauma	205 (64%)
Burn	12 (3.75%)
ARDS	26 (8.12%)
Sepsis	48 (15%)
Postsurgical complications	29 (9.13%)
Body mass index (kg/m^2^)	27.2 ± 10.3
Anatomic vascular abnormality (%)	6 (1.87%)
Untreated coagulopathy (%)	0 (0%)
Prophylactic treatment with LMWH (%)	320 (100%)
Hypercoagulable state (%)	16 (5%)
Malignancy (%)	23 (7.18%)
Total number of UEDVT (%)	36 (11.25%)
Central venous catheters	177 (55.3%)
Other intravascular devices	16 (5%)
Days of hospitalization	59 ± 26

Abbreviations are: APACHE II: acute physiology and chronic health evaluation score; ARDS: acute respiratory distress syndrome; LMWH: low molecular weight heparin; UEDVT: upper extremity deep venous thrombosis.

**Table 2:** Characteristics of the 36 cases with upper extremity deep venous thrombosis (UEDVT).

Characteristics	Number (percent)
*Location of UEVT*	
Internal jugular vein	25
Subclavian vein	27
Innominate vein	9
Axillary vein	11
Brachial	6

*Number of venous segments involved*	
Single segment	14
Two segments	12
Three segments	10
Four segments	4
Five segments	2

*Clinical characteristics*	
Spontaneous thrombosis	3 (8.3%)
Catheter-associated thrombosis	33 (91.7%)*
Triple-lumen catheter	14 (38.8%)
Hemodialysis (double-lumen catheter)	19 (52.7%)
Malignancy	14 (38.8%)
Hypercoagulable state	8 (22.2%)
Total parenteral nutrition	9 (25%)
Body mass index ≥35 kg/m^2^	8 (22.2%)
Asymptomatic thrombosis	16 (44.4%)
Symptomatic thrombosis	20 (55.6%)
Subsequent pulmonary embolism	2 (5.5%)
ICU deaths	1 (2.7%)
Therapeutic anticoagulation	36 (100%)

*General sonographic characteristics*	
Acute thrombosis	28 (77.8%)**
Chronic thrombosis	8 (12.2%)

*Catheter-associated versus spontaneous thrombosis and

**acute versus chronic thrombosis (both *P* < 0.01; Fisher's test).

**Table 3:** Estimating the relative age of venous thrombus by ultrasound.

Characteristics	Number/total cases
*Chronic thrombosis (n* = 8)	
Contracted venous segment	6/8
Clot adherence	8/8
Free-floating thrombi	1/8
Hyperechoic thrombi	4/8
Homogeneous thrombi	1/8
Partial recanalization	7/8
Venous collaterals	7/8
*Acute thrombosis (n* = 28)	
Venous distention	22/28
Lumen partially and/or noncompressible	26/28
Hypoechoic thrombi	18/28
Homogeneous thrombi	22/28
Free-floating thrombi	20/28
Double hyperechoic line	20/28
along the thrombus/wall interface	

**Table 4:** Multivariate logistical regression correlating various parameters with the incidence of upper extremity deep venous thrombosis.

Effect	Odds ratio estimates		
	Point estimate	95% confidence limits	*P*
Central venous catheter	2.716	2.312–2.911	0.007
Triple lumen catheter	1.515	1.108–2.166	0.035
Hemodialysis (double-lumen) catheter	1.823	1.245–2.344	0.024
Malignancy	1.483	1.107–1.746	0.036
Total parenteral nutrition	1.399	1.066–1.699	0.042
Hypercoagulable state	1.284	1.108–1.382	0.045
Body mass index ≥35 kg/m^2^	1.191	1.079–1.402	0.049
